# Reanimation of Stored Tissue Biopsies: A Functional Study and Translational Approach

**DOI:** 10.3390/ijms27031298

**Published:** 2026-01-28

**Authors:** Veronica Alfano, Gabriele Ruffolo, Antonella Spila, Maria Giovanna Valente, Luigi Sansone, Manuel Belli, Dania Ramadan, Chiara Miele, Luca Garelli, Leonardo Lupacchini, Patrizia Ferroni, Daniela Merlo, Eleonora Palma, Fiorella Guadagni

**Affiliations:** 1 IRCCS San Raffaele Roma, 00166 Rome, Italy; veronica.alfano@sanraffaele.it (V.A.); gabriele.ruffolo@uniroma1.it (G.R.); antonella.spila@sanraffaele.it (A.S.); maria.valente@sanraffaele.it (M.G.V.); luigi.sansone@uniroma5.it (L.S.); manuel.belli@uniroma5.it (M.B.); dania.ramadan@sanraffaele.it (D.R.); kiara.m.94@hotmail.com (C.M.); luca.garelli@sanraffaele.it (L.G.); patrizia.ferroni@uniroma5.it (P.F.); 2 Department of Human Sciences and Promotion of the Quality of Life, San Raffaele Roma University, Via di Val Cannuta, 247, 00166 Rome, Italy; fiorella.guadagni@uniroma5.it; 3 Department of Physiology and Pharmacology, Sapienza University of Rome, 00185 Rome, Italy; eleonora.palma@uniroma1.it; 4 Department of Neuroscience, Istituto Superiore di Sanità, 00161 Rome, Italy; daniela.merlo@iss.it; 5 InterInstitutional Multydisciplinary Biobank (BioBIM), IRCCS San Raffaele Roma, 00166 Roma, Italy

**Keywords:** functional study, ion channels, microtransplantation, *Xenopus* oocyte model, translational approach, cancer

## Abstract

The availability of biobanked tissues represents an important resource for translational research; however, functional investigations are generally limited to freshly collected samples. To address this limitation, we developed an innovative strategy to restore functional properties of frozen biopsies by microtransplanting patient-derived membrane proteins into *Xenopus laevis* oocytes. This study aimed to recover and characterize the physiological properties of human colon cancer cell membranes and to investigate the role of neurotransmitter-related signaling and ion currents in cancer. Membrane incorporation was assessed by immunohistochemical detection of tumor-specific markers, including carcinoembryonic antigen, together with confocal microscopy and ultrastructural analyses. Functional viability was evaluated using two-electrode voltage clamp recordings to assess endogenous calcium-activated chloride currents and responses to selected neurotransmitters. The successful incorporation of colon cancer membranes was confirmed by specific immunoreactivity and ultrastructural features consistent with cancer cell architecture. Although no functional responses to the tested neurotransmitters were detected, oocytes microinjected with cancer membranes showed a marked reduction or complete suppression in endogenous calcium-activated chloride currents. These findings demonstrate that membrane microtransplantation into *Xenopus* oocytes is a reliable translational approach to functionally investigate cancer cell membranes from frozen biopsies, and suggest that altered chloride channel activity may represent a baseline for new studies to investigate new potential therapeutic targets for colon cancer.

## 1. Introduction

Innovative experimental strategies are required to extend the translational value of biobanked human tissues, which remain largely underexploited because of the loss of functional integrity following long-term storage. A major limitation is the inability to perform functional analyses on frozen specimens, which has restricted the study of pathophysiological mechanisms in cancer.

Just to give an example, ion channels are pivotal regulators of cellular physiology, contributing to neurotransmission, signal transduction, modulation of the cell cycle, and many other biological processes [1]. Accordingly, their role in cancer development and progression is under active investigation, as targeting channel-mediated signaling may provide additional therapeutic opportunities [1]. However, electrophysiological investigations on cancer tissues are not routinely performed, and technical constraints currently prevent the systematic use of frozen tissue biopsies, the most abundant and accessible source of patient-derived cancer material.

To address these unmet needs, we focused on one of the most clinically and epidemiologically relevant cancer types, colorectal cancer, whose incidence is steadily increasing worldwide [2], and for which electrophysiological alterations have been previously documented [3,4,5]. However, although its pathogenesis and the contribution of the inflammatory milieu have been extensively characterized [2], the potential involvement of electrophysiological processes remains poorly understood. Indeed, while abundant evidence documents the activity of neurotransmitter receptors in nervous system tumors and the alterations of inhibitory and excitatory signaling systems [6,7,8,9], the role of these mechanisms in non-neurological cancers remains unclear.

Increasing evidence suggests that neurotransmitters or neuromodulators may contribute to colorectal cancer onset [3]. Several studies have reported aberrant expression of neurotransmitter signaling genes in growing and/or progressing tumors, supporting the hypothesis that these pathways may promote proliferation, migration, invasion, and angiogenesis. In particular, in colorectal cancer, neurotransmitters may also interact with immune and endothelial cells in the tumor microenvironment to promote inflammation and progression, although the underlying pathophysiological basis remains unresolved [3]. Furthermore, both GABA_A_ and GABA_B_ receptors have been implicated in either pro- or anti-tumorigenic responses in this type of cancer [4]. Ion channels are also recognized as key regulators of proliferation, migration, and apoptosis [4], and dysregulation of calcium-activated chloride channels has been associated with colorectal cancer progression [10], with their downregulation correlating with inflammatory components that facilitate tumor invasion in the gastrointestinal tract [5].

In this context, our study proposes an innovative, translational, and methodological approach based on the analysis of cell membranes extracted from different types of cancer samples, including a patient biopsy and cell lines, collected and stored in liquid nitrogen. This strategy enables the “reanimation” of frozen patient-derived cancer tissues in *Xenopus* oocytes, providing a unique opportunity to reutilize otherwise inaccessible clinical samples. Furthermore, by characterizing in vitro the molecular and functional properties of cancer ion channels, this model allows the investigation of pathophysiological mechanisms in non-neurological cancers, such as colon and pancreatic cancer. Ultimately, this methodological framework and translational approach aim to maximize the scientific value of precious frozen clinical specimens to carry out studies aimed at identifying potential diagnostic/therapeutic targets for cancer or other chronic disorders.

## 2. Results

First, we evaluated the effect of cancer cell membranes microtransplantation on *Xenopus* oocytes in terms of cell survival. To this purpose, we performed a mortality test on the microtransplanted oocytes at different time points after colon cancer cell membrane injection (24 and 48 h). These time points were selected based on previous experiments that demonstrated that peak membrane expression of injected proteins in the oocytes is reached in the time interval between 24 and 48 h after cytoplasmic injection [11]. We found that cancer cell membranes injection determined a 90% mortality at 24 h (Patient 1; 4 of 40 live cells obtained from 2 frogs). Similarly, a 96.3% mortality (Patient 1; 5 of 136 live cells obtained from four frogs) was observed after 48 h. This phenomenon was probably due to toxic components in the cancer cell membranes, as the mortality rate was significantly higher (both at 24 and 48 h) when compared to oocytes that were injected with Gly buffer only (sham injection, 35 of 40 live cells (Patient 1; 2 frogs) at 24 h, *p* < 0.001, chi-squared test; 33 of 41 live cells (Patient 1; 4 frogs) at 48 h, *p* < 0.001, chi-squared test).

Hence, to prevent cell mortality and to define the optimal membrane dilution to use in further experiments, we repeated the mortality assay with progressive dilutions of the original colon cancer membrane samples (50%, 16.7%, and 4.2%). Using this approach, we found a progressively lower mortality rate (Table 1).

### 2.1. Immunohistochemistry and Immunofluorescence

**Figure 1:**
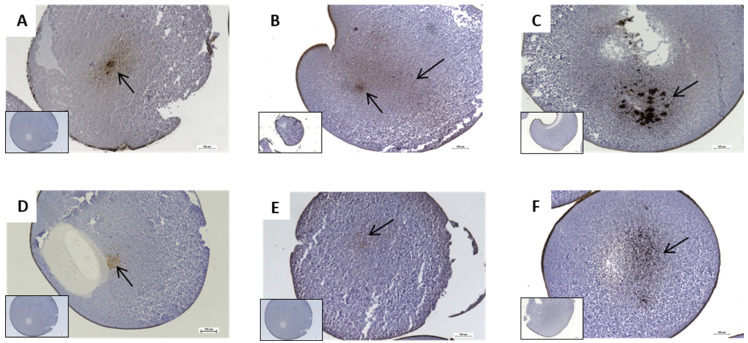
Representative images of IHC-stained sections of *Xenopus laevis* oocytes injected with fully processed cell membranes: (**A**) CEA (COL-1) staining of colon adenocarcinoma tissue fragments; (**B**) cytokeratin (CAM 5.2) staining of HT-29 microinjected membranes; (**C**) E-Cadherin staining of HT-29 microinjected membranes; (**D**) CEA (COL-1) staining of HT-29 microinjected membranes; (**E**) CEA (COL-1); and (**F**) E-Cadherin staining of microinjected membranes from ascitic pancreatic tumor cells. Areas of observed immunoreaction are indicated by arrows. The inset image shows the absence of staining in non-injected oocytes (negative control). Magnification 100×. (scale bar 100 µm).

To further determine whether frozen tumor tissue remains viable for functional studies, we injected fully processed membranes from a colon cancer tissue biopsy, the HT-29 human colorectal adenocarcinoma cell line, and a metastatic pancreatic ascites primary cancer cell line into *Xenopus laevis* oocytes. We then performed immunohistochemical staining using a panel of tumor membrane biomarkers (see Table 2). The results showed that, despite freezing, the tumor cell membranes retained the ability to express relevant markers (Figure 1).

**Figure 2:**
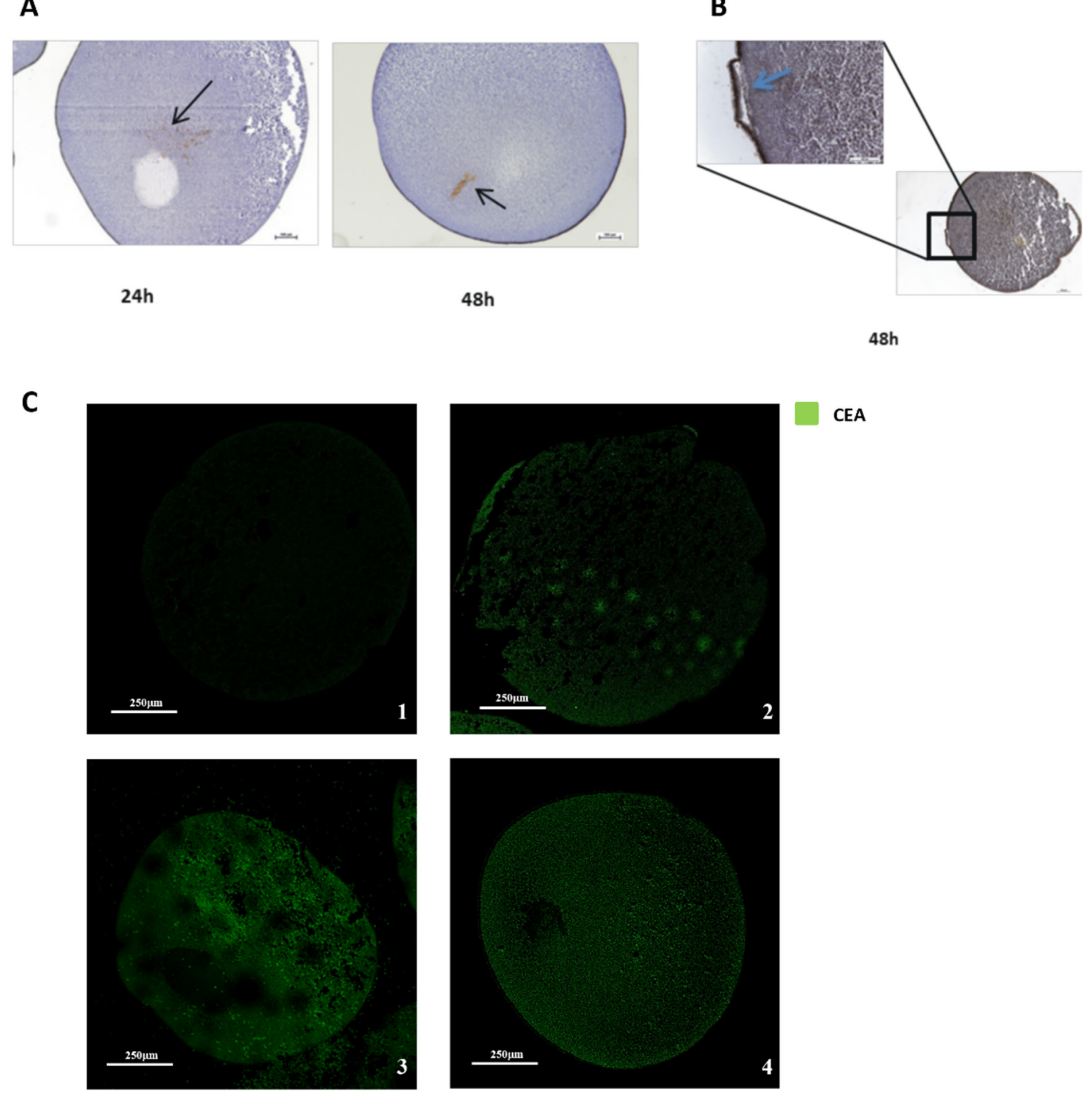
(**A**) Representative microphotographs of time-course microtransplantation of processed HT-29 cell membranes at 24 h and 48 h. The black arrows indicate the localization of the membrane in the *Xenopus laevis* oocytes tested at different times from the injection. Antibody [COL-1], magnification 100×. (scale bar 100 µm) (**B**) The immunohistochemical expression of tumor tissue membrane fragments at the level of the oocyte membrane, which was camouflaged by the melanic staining of the frog oocyte animal pole (blue arrow). (scale bar 100 µm and 250 µm) (**C**) A confocal microscopy image (magnification 60×) showing, by CEA expression, the distribution of membranes in the microtransplanted *Xenopus* oocytes. Negative control (1); cancer tissue cell membranes (2); HT-29 cell membranes (3); metastatic pancreatic ascites cancer cell membranes (4); (scale bar 250 µm).

**Figure 3:**
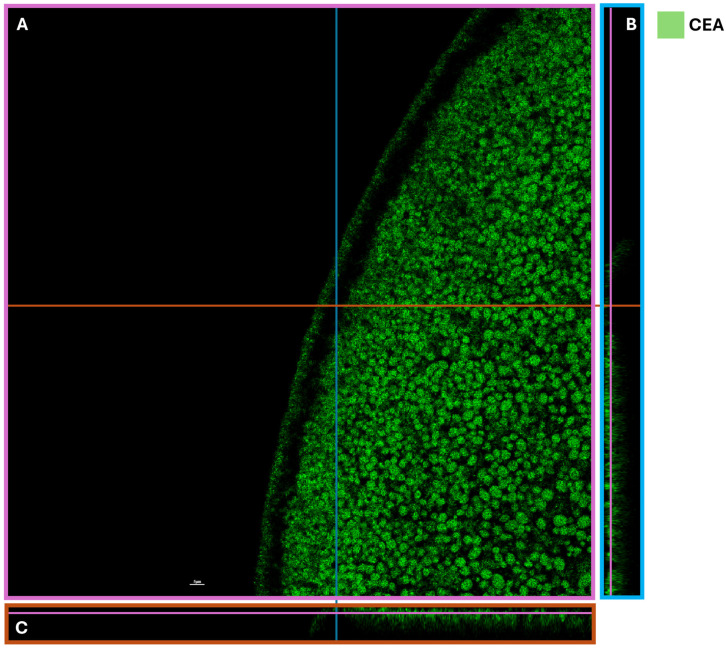
A representative confocal microscopy image of the injected *Xenopus* oocyte at 60× magnification: (**A**) the side view of the z-stack, x and y planes, pink projection; (**B**) the side view of the z-stack and y and z planes, blue projection; and (**C**) the side view of the z-stack and x and z planes, orange projection. The Z-stack images show CEA expression (indicated in green) on the cancer cell membranes localized on the oocyte surface and in peripheral cytoplasmic regions; (scale bar 5 µm).

In addition to visualizing the immunological integrity of the cell membranes, we also analyzed the distribution and movement of the injected material within the *Xenopus laevis* oocyte. To investigate its migration, we conducted a time-course study at 24 and 48 h from injection using an anti-carcinoembryonic antigen antibody [COL-1], which is expressed in both colon cancer tissue and the HT-29 cell line. Oocytes were injected with membranes derived from a colon cancer biopsy and HT-29 cells. At 24 h post-injection, we observed an accumulation of cancer material near the injection site. After 48 h, the material had migrated toward the oocyte membrane (Figure 2A). Specifically, CEA was detected at the oocyte membrane, visually distinguishable against the pigmented animal pole of the *Xenopus laevis* oocyte (Figure 2B). These analyses demonstrated the presence of membrane fragments at the oocyte surface but did not clarify whether the fragments remained within the cell or were released externally. For this purpose, we performed immunofluorescence staining using the same anti-CEA antibody and examined the samples with confocal microscopy. The results confirmed the specificity of the cancer material and the presence of HT-29 cell membranes within the oocytes (Figure 2C). Confocal z-stack acquisition showed tumor fragments localized specifically at the oocyte membrane, with no detectable fragments outside the oocyte (Figure 3). This confirms that the injected material remains confined to the oocyte membrane.

### 2.2. Transmission Electron Microscopy (TEM)

**Figure 4:**
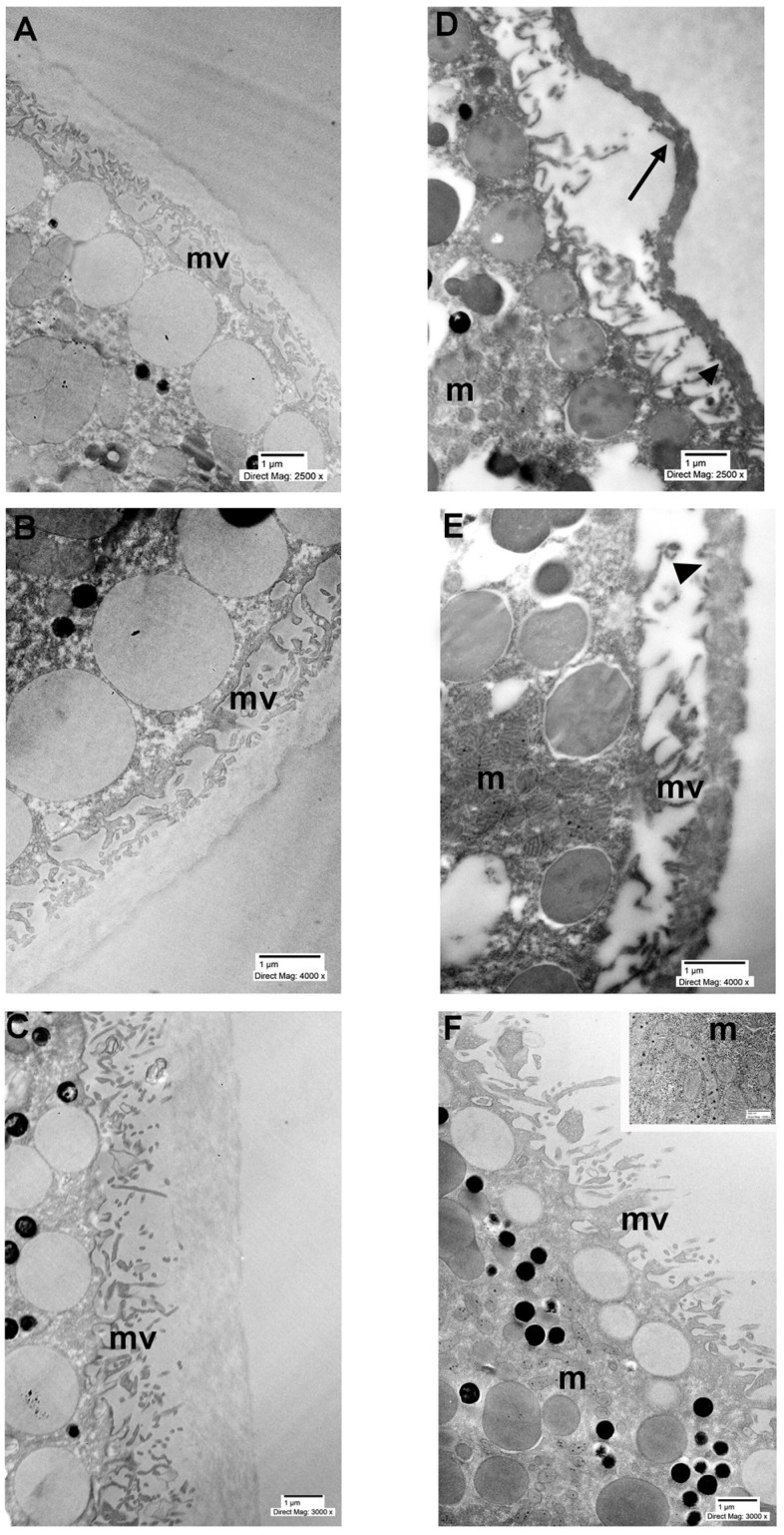
Ultrastructural evaluation of control (**A**–**C**) and microtransplanted oocytes (**D**–**F**) at 48 h after injection. (**A**) TEM micrograph of control oocyte showing a clear and intact external membrane surrounding the ooplasm (TEM bar: 1 µm). (**B**) High magnification of control oocyte external membrane (TEM bar: 1 µm). (**C**) Detail of an intact membrane from the control group (TEM bar: 1 µm). (**D**) TEM micrograph of microtransplanted oocyte (colon cancer tissue) showing a high electron-dense membrane with evident signs of detachment (TEM bar: 1 µm). (**E**) High magnification of the microtransplanted oocyte (HT-29 human colorectal adenocarcinoma) external membrane, presenting several areas of thinning. (**F**) TEM picture of the metastatic pancreatic ascites cancer cells microtransplanted oocyte showing the absence of the membrane. Inset F: high magnification of healthy mitochondria showing numerous cristae. mv: microvilli; m: mitochondria; arrow: membrane detachment; arrowhead: invagination.

TEM evaluation of the control group (non-microtransplanted oocytes) revealed that the plasma membrane appeared as a continuous structure with a typical unit membrane profile. The membrane exhibited a smooth contour with occasional invaginations, and numerous microvilli were present. Beneath the plasma membrane, prominent yolk platelets and cortical granules were observed. In some regions, the membrane was closely associated with underlying components of the cytoskeleton (Figure 4A–C). In the treated group (oocytes microtransplanted with cell membranes from a patient’s colon cancer biopsy and HT-29 cells), the plasma membrane appeared more electron-dense and thicker, not like the one observed in the control result. Additionally, we observed membrane thinning in several areas. Detachment of the plasma membrane from the oolemma was also evident. The microvilli underlying the membrane appeared fewer and thinner (Figure 4D,E). The oocytes microtransplanted with metastatic pancreatic ascites cancer cell membranes, unlike the control and the other experimental groups, showed areas without the presence of the exogenous membranes. Despite this appearance, the ooplasm was well preserved, with numerous healthy mitochondria displaying abundant cristae (Figure 4F, inset).

### 2.3. Electrophysiological Study on Xenopus Oocytes Microtransplanted with Cell Membranes from Colon Cancer Biopsy and Cancer Cell Lines

In the first set of experiments, we tested the response of oocytes microtransplanted with colon cancer cell membranes diluted at 50% (dil 1:2 with membrane buffer) to different neurotransmitters, 48 h after injection at different holding potentials (−60 mV, −80 mV, and −100 mV) obtained from three frogs and Patient 1. Despite the different neurotransmitters tested (GABA, ACh, ATP-Mg^2+^, Gly, and Glu, at concentrations ranging from 0.1 to 1 mM), we could not record any response in our experimental conditions. As positive controls, in the same experiments, we observed valid neurotransmitter GABA- ACh-, Gly- Glutamate-evoked currents from oocytes injected with TLE or ALS tissues, which we already tested in previous experiments (Supplementary Table S1).

**Figure 5:**
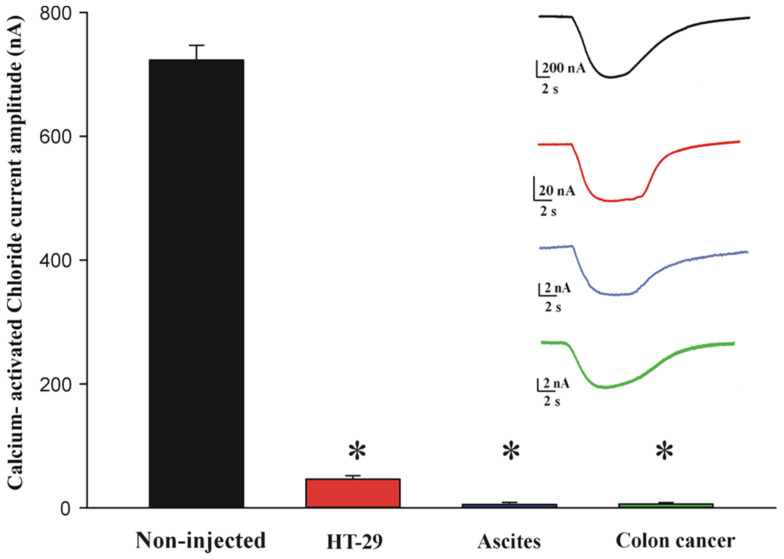
The calcium-activated chloride currents study on the cancer cell membranes. The bar graph shows the mean of the calcium-activated chloride current amplitude (nA) recorded on oocytes microtransplanted with HT-29 cell line membranes (red), Patient 4 pancreatic cancer ascites cell line membranes (blue), and Patient 1 colon cancer membranes (green), compared with non-injected cells as a control (black) from 2 frogs. Data are expressed as mean ± s.e.m; * = *p* < 0.01 by Student’s *t*-test. Inset: representative chloride current traces for each group studied.

In another set of experiments, we focused on calcium-activated chloride currents. As previously reported [12], the injection of a calcium chloride solution (50 mM) in naïve *Xenopus* oocytes yields large chloride currents (723.2 ± 23.7; range: 332.3–964.8 nA; n = 12). We found that this current was greatly diminished when the oocytes were microtransplanted with cells from the human colon cancer cell line HT-29 (46.2 ± 5.6 nA; range 21.2–69.1 nA; n = 11; *p* < 0.01, Student’s *t*-test), and almost completely abolished when an ascites cell line and a colon cancer biopsy were used for the microtransplantation (5.4 ± 3.3 nA; range: 1.7–8.4 nA; n = 3, ascites cell line; 6.4 ± 2.3 nA; range: 1.5–9.3 nA; n = 3, colon cancer tissue; *p* < 0.01, Student’s *t*-test; see Figure 5). The results at 48 h post-injection were obtained from the oocytes harvested from two frogs and two patients (1 and 4).

#### Electrophysiological Responses in Tumor Tissues Compared to Corresponding Normal Mucosa

**Figure 6:**
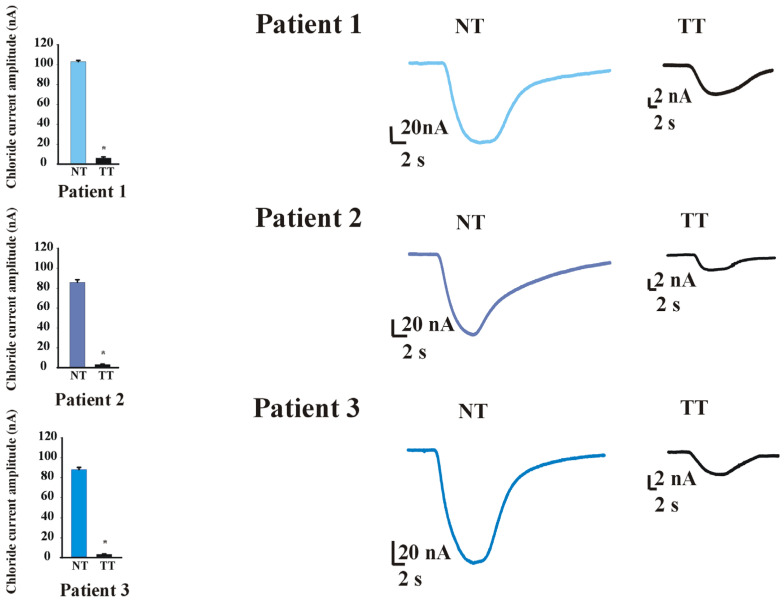
Calcium-activated chloride currents study on cancer cell membranes. The bar graphs on the left show the mean of the calcium-activated chloride current amplitude (nA) recorded on the oocytes microtransplanted with three different colon tumor tissues (TTs) and their corresponding normal colonic mucosa (NT). The right panels show representative chloride current traces for each group studied. The data are expressed as mean ± s.e.m; * = *p* < 0.001 by Student’s *t*-test.

A dedicated set of experiments was designed to evaluate calcium-activated chloride currents in oocytes microtransplanted with the membranes of three different colon cancer tissues, with their corresponding histologically confirmed normal colonic mucosa. As previously reported [13], the injection of a calcium chloride solution (50 mM) in *Xenopus* oocytes with their corresponding normal colonic tissues yields large chloride currents (NT, 93.8 ± 2.7 nA; range: 79.0–108.0 nA; n = 13; Patients 1–3; two frogs; see Figure 6). We found that this current was greatly diminished when the oocytes were microtransplanted with cells from human colon cancer (TT, 4.3 ± 0.8 nA, range: 1.5–9.3 nA, n = 14; Patients 1–3; two frogs; *p* < 0.001, Student’s *t*-test; see Figure 6). The results obtained for each individual patient are shown in Figure 6 and Table 3.

## 3. Discussion

Here, we propose an innovative and translational methodological approach to recover and study the functional properties of tumor proteins from biobanked cancer patient biopsies, with the objective of increasing the knowledge of the mechanisms underlying cancer and identifying new therapeutic targets. Specifically, we applied membrane microtransplantation to investigate the functional properties of cell membranes extracted from cancer cells and tissues and microinjected into *Xenopus* oocytes.

There are two main strengths that lie in the use of this method in this field of investigation. First, we propose a procedure that allows us to “reanimate” antigens from frozen cancer specimens, thus enabling their study in a living cell, with all the experimental advantages that this implies. Second, we set up a system that allows us to record electrophysiological responses from cancer tissues when present.

Specifically, our main findings were: (i) cancer membranes can be successfully isolated and microtransplanted in *Xenopus* oocytes, both from cell lines and biopsies; (ii) it is possible to find and study cancer-specific antigens (such as CEA) onto the outer membrane of the oocytes by means of immunohistochemical techniques; and (iii) even though we could not record membrane currents after the application of several among the most relevant neurotransmitters, we found that *Xenopus* oocytes endogenous calcium-activated chloride currents are diminished or totally abolished by the injection of cancer membranes from cell lines or biopsy specimens.

Our findings demonstrated, in particular, that these cancer membranes were still able to express pertinent markers even after prolonged freezing times. These results were further supported by confocal microscopy analysis of immunofluorescence staining with the same anti-CEA antibody used in immunohistochemical studies. Moreover, the TEM images acquired on the microinjected *Xenopus* oocytes revealed distinct ultrastructural alterations. In particular, while in the control group the plasma membrane appeared continuous with a typical bilayer structure and numerous microvilli, the treated group exhibited a thickened, higher electron density with discontinuities and reduced microvilli. This is consistent with previous studies reporting altered membrane architecture in cancer cells, often associated with increased stiffness, disrupted cytoskeletal anchoring, and impaired membrane dynamics [12,13]. To the best of our knowledge, this is the first study reporting architectural data on microinjection of cell membranes from frozen colon cancer tissues in *Xenopus* oocytes. However, it should be noted that TEM was performed to gain more descriptive information on this in vitro model. Specifically designed studies should be conducted to further investigate the oocyte response to cancer cell membrane incorporation. Interestingly, the oocytes microtransplanted with metastatic pancreatic ascites cancer cells showed the absence of the vitelline membrane and a well-preserved ooplasm. These findings may suggest a biologically relevant interaction between the injected cancer material and the oocyte’s extracellular environment. In *Xenopus*, the vitelline membrane is a glycoprotein-rich layer that plays a crucial role in protecting the oocyte and mediating sperm recognition during fertilization [14]. Its partial absence in this context, however, may reflect a favorable remodeling process or a structural adaptation triggered by the presence of specific cancer-derived factors. Furthermore, the preserved mitochondrial morphology further suggests that the oocytes retained metabolic activity and were not subjected to stress or damage severe enough to impair their viability. On the other hand, we acknowledge that our ultrastructure analysis yields qualitative observations that demand further investigation.

In addition, our study showed that the microtransplantation of tissue membranes from different sources (cancer biopsies or cell lines) under appropriate experimental conditions does not have a negative impact on oocyte viability. This opens significant perspectives for the use of our approach for wider applications in cancer studies.

Contrary to our expectations, even though we demonstrated that the injected material successfully relocates onto the oocyte’s outer membrane, we did not record any ligand-gated receptors’ response after the application of GABA, ACh, ATP-Mg^2+^, Gly, and Glu. On one hand, this finding apparently conflicts with published evidence that puts neurotransmitter systems under the spotlight as adjunctive mediators of colon cancer onset and progression [4,15,16], but on the other hand, it could suggest that these biological effects may not be linked with ionic current activation, but with alternative signaling pathways that follow the binding of the ligands to their receptors.

Calcium-activated chloride channels have also been shown to play a significant role in carcinogenesis and progression. In particular, a negative regulation of their function has been correlated with the processes of onset and generation of colon cancer [5]. In this framework, our results strengthen this hypothesis and suggest that cancer membranes incorporate factors that contribute to the inhibition of calcium-activated chloride channels, while, notably, in healthy mucosal tissue, calcium-activated chloride currents are preserved, a result that is in line with our previous observations using brain tissues [13]. Interestingly, we also described that the magnitude of this phenomenon varies depending on the type of cancer material that is injected into the oocytes. Based on this observation, we can hypothesize that the “inhibiting capacity” of cancer membranes on chloride currents may depend on the differentiation state of the cell lines/cancer tissues that are used for the microinjection. This would suggest, in line with previous observations [5,10], that cancer progression at later stages correlates with a higher degree of inhibition of this calcium activated chloride currents.

Indeed, neurotransmitter receptors and chloride-activated calcium channels are not the only relevant phenomena that could be studied with our approach. Recent evidence also highlights the role of specific transporter proteins [17] and/or voltage-gated ion channels [18]. While we did not tackle these issues in the present work, their study using our method represents an interesting perspective, nonetheless, as microtransplantation allows us to also record the electrical activity stemming from the function of these membrane proteins [19,20]. Moreover, further refinement of this model through translational approaches could yield a better personalized care pathway not only for cancer patients, but also for other complex chronic disorders (i.e., neurodegenerative diseases).

In summary, our study proposed an innovative methodological approach that utilizes the microtransplantation of cancer-derived cell membranes into *Xenopus* oocytes, combined with advanced ultrastructural and electrophysiological analyses. This strategy made possible the functional “reanimation” of bioptic tissue material, providing unique insight into membrane-associated phenomena—including receptor response and ion channel modulation—in a living system. We recognize that we did not describe the mechanistic substrate of this phenomenon, as this study was primarily focused on setting up a method to incorporate and study biobanked colon cancer tissues in *Xenopus* oocytes. Hence, the pathophysiological implications of this study will be the object of future investigation. Although the present work focused mainly on neurotransmitter receptors and calcium-activated chloride channels, the potential of this method opens up promising avenues to investigate additional membrane proteins, such as transporters and voltage-gated channels, in future studies. Hence, the ability to recover and study the physiological properties of cryopreserved and otherwise non-viable tissues represents a useful tool to expand knowledge of cancer disease mechanisms and progression.

## 4. Materials and Methods

### 4.1. Patients and Sample Collection

Experiments were performed on membrane extracts obtained from frozen colon cancer surgical biopsies (Tumor Tissue = TT) and corresponding histologically confirmed normal mucosa (Normal Tissue = NT) from three male patients (patient codes: BIOBIM-201001750 Patient 1, BIOBIM-201003259 Patient 2, and BIOBIM-201003705 Patient 3, aged 65, 61, and 64 years, respectively). Two patients (1 and 2) had left-sided colon adenocarcinoma, and patient 3 had right-sided colon adenocarcinoma. All patients underwent video-laparoscopic colon resection. Experiments were also performed on an established colon cancer cell line (HT-29) and on a primary cell line derived from pancreatic malignant ascites obtained from one female patient (BIOBIM-202400051 Patient 4, 50 years old) with pancreatic mucinous adenocarcinoma. The surgical specimens and cell lines included in this study were provided by the Interinstitutional Multidisciplinary Biobank of the IRCCS San Raffaele (SR-BioBIM, Rome, Italy), a Biological Resource Center formally approved by the local Ethic Committee (approval number ISR/DMLBA/405). Before participating in the SR-BioBIM project, all donors are adequately informed by a physician about the importance of biobanks for the purposes of scientific research and the reasons why biological samples are collected; the informed consent form is signed by both parties. This study was performed in accordance with the principles embodied in the Declaration of Helsinki and guidelines of the Italian Ministry of Health (no. Authorization 427/2020-PR).

### 4.2. Mortality Assay

Cell mortality experiments were performed to assess the viability of microtransplanted oocytes. Two complementary approaches were employed: morphological analysis under an optic microscope and electrophysiological measurements of the resting membrane potential (RP). The oocytes were observed at 24 and 48 h from microtransplantation under an optic microscope, and classified as viable based on morphological integrity, such as well-defined shape and the absence of cell lysis or swelling. Oocytes showing membrane rupture, swelling, or abnormal morphology were considered non-viable [21]. In parallel, the RP was measured with intracellular microelectrodes using a voltage clamp setup in the oocyte cytoplasm and recorded under steady-state conditions after 10–15 min of stabilization. A depolarized RP was indicative of compromised membrane integrity and was associated with early apoptotic events in *Xenopus* oocytes [21,22]. Hence, oocytes with evident white spots and with RP > −35 mV were considered not viable and discarded. Conversely, RP values within the physiological range for these cells [23] (–40 to –60 mV) were associated with viable oocytes, which were used in subsequent experiments. The control oocytes, not subjected to membrane microtransplantation, but kept under the same experimental conditions, were used to establish baseline RP values and morphological integrity.

### 4.3. Membrane Preparation and “Two-Electrode Voltage-Clamp Recordings”

The procedure of membrane extraction from human tissues or cell lines was previously described in detail [11]. Briefly, samples were homogenized with a Teflon glass homogenizer using 2 mL of glycine buffer (composition in mM: 200 glycine, 150 NaCl, 50 EGTA, 50 EDTA, and 300 sucrose; plus 20 μL protease inhibitors [P2714; Sigma, Darmstadt, DE]; pH 9 adjusted with NaOH). Subsequently, the homogenate underwent two centrifugation cycles, the first at 9500× *g* for 15 min in a Beckmann centrifuge (C1015 rotor; Palo Alto, CA, USA). Afterwards, the supernatant was collected and subjected to a second centrifugation at 100,000× *g* for 2 h in a TL-100 rotor at 4 °C. The EGTA/EDTA are washed away after the membrane extraction process, as this is a part of the membrane extraction from tissues and does not interfere with the experimental procedures on the oocytes. The pellet was washed, re-suspended in assay buffer (glycine 5 mM), and used directly, or aliquoted and stored at −80 °C for further use. The buffer composition at the moment of injection is a sterile bi-distilled water solution with 5 mM glycine. Preparation of *Xenopus laevis* oocytes and injection procedures are described elsewhere [11,24]. The oocytes were injected with about 100 nL membrane preparation (0.2–10 mg/mL of total protein) [11,25]. The use of female *Xenopus laevis* frogs conformed to the institutional policies of Sapienza University of Rome and guidelines of the Italian Ministry of Health (no. Authorization 427/2020-PR). Voltage-clamp experiments on *Xenopus* oocytes were performed at 24 h and 48 h after injection using two microelectrodes filled with 3M KCl. During the recordings, the oocytes were placed in a recording chamber (volume, 0.1 mL) and perfused continuously (9–10 mL/min) with the oocyte’s Ringer solution (OR, composition in mM: 82.5 NaCl; 2.5 KCl; 2.5 CaCl_2_; 1 MgCl_2_; 5 HEPES, adjusted to pH 7.4 with NaOH) at room temperature (20–22 °C). Unless otherwise specified, holding potential was held at −60 mV during each recording [26]. For some experiments, the colon cancer membranes were diluted using a membrane buffer (glycine 5 mM) as follows (expressed as the ratio between the membrane sample volume and total volume): 1:2 (50%), 1:6 (16.7%), and 1:24 (4.2%). Chemicals and neurotransmitters used were gamma-aminobutyric acid (GABA), acetylcholine (ACh), ATP-magnesium complex (ATP-Mg^2+^), glycine (Gly), and glutamate (Glu), all purchased from Tocris Bioscience (Bristol, UK). Each of them was dissolved in sterile water, stored as a frozen stock solution, and diluted to the Ringer solution (OR) working concentration before each recording session. In a set of experiments, we measured the Cl^−^ reversal potential (E_Cl_) under different conditions. Calcium chloride (${CaCl}_{2}$) was pressure-injected into oocytes from a pipette containing 50 mM ${CaCl}_{2}$ [12,13].

### 4.4. Immunohistochemistry

Paraffin-embedded tissue blocks were prepared from microtransplanted *Xenopus laevis* oocytes and sectioned into 4 μm slices. The membranes extracted from a colon cancer biopsy for this set of experiments were diluted in a ratio of 1:2 with membrane buffer (Gly 5 mM). Cancer membrane distribution in microtransplanted oocytes was assessed by immunohistochemical staining using the antibodies listed in Table 2.

Immunohistochemical staining was performed using an EnVision™ FLEX + Mouse in the Dako Autostainer Detection System (Dako-Agilent, Santa Clara, CA, USA), according to the manufacturer’s protocol with proprietary reagents. Briefly, after pre-treatment with heat-induced epitope retrieval (HIER), slides were treated with Dako Block buffer 3% H_2_O_2_-HRP (EnVision FLEX Peroxidase-Blocking Reagent, Dako-Agilent) for 5 min to block endogenous peroxidase activity. Subsequently, the primary antibodies were incubated at RT for 40 min. The HRP-linked secondary antibody was incubated at RT for 15 min (EnVision FLEX/HRP, Dako, Glostrup, Denmark). Slides were washed 3 times for 5 min each in a Wash buffer between incubations. HRP activity was detected using Dako Liquid DAB + Substrate Chromogen System (DAB Flex, Dako-Agilent) for 10 min. Tissue sections were then counterstained with hematoxylin (Hematoxylin Flex, Dako, Glostrup, Denmark) for 5 min. At the end of the procedure, after dehydration with alcohol/xylene baths, the tissue sections were stabilized with mounting medium (Eukitt, Merck KGaA, Darmstadt, Germany). Standardized sections were included as positive controls, whereas sections incubated without primary antibody were used as negative controls.

### 4.5. Immunofluorescence

Immunofluorescence staining was carried out on formalin-fixed, paraffin-embedded tissue sections from *Xenopus* oocytes. Slides were deparaffinized, rehydrated, and subjected to HIER using Tris/EDTA buffer (pH 9; EnVision FLEX Target Retrieval Solution, High pH, Dako-Agilent) in a Dako PT Link system.

Slides were incubated for 5 min with 3% H_2_O_2_ (EnVision FLEX Peroxidase-Blocking Reagent, Dako-Agilent) and then with primary antibody CEA (National Institutes of Health, Bethesda, MD, USA), properly diluted using EnVision™ FLEX antibody diluent (Dako-Agilent), for 40 min at room temperature. Sections were then incubated with Alexa Fluor 488-conjugated goat anti-mouse IgG1 secondary antibody (Invitrogen, Waltham, MA, USA) for 1 h at 37 °C. Slides were mounted with aqueous mounting medium, Fluoromount (Merck KGaA, Darmstadt, Germany). Images were acquired in 2D and z-stack, MIP [27,28], using a Nikon Eclipse Ti2-A1 RHD25 Confocal Microscopy System with NIS-Elements AR 5.20.02 software (Nikon Europe B.V., Amstelveen, The Netherlands).

### 4.6. Transmission Electron Microscopy

The samples, after different treatments, were collected and fixed overnight in 2.5% glutaraldehyde with 0.1 M sodium hydroxide, 0.1 M, pH 7.3. The samples were washed 6 times in sodium hydroxide buffer, and then post-fixed in 2% osmium tetroxide in the same buffer for 2 h at room temperature and treated following a standard protocol for embedding in EPON resin [29]. Next, a polymerization procedure overnight at 65 °C was performed. Ultrathin sections of 80 nm of thickness were cut on a Leica Ultracut E Ultramicrotome (Leica Microsystems, Wetzlar, Germany) and placed on copper grids, contrasted with UranyLess stain and lead hydroxide, and, lastly, examined in a JEOL-1400 Plus TEM (Jeol Ltd., Tokyo, Japan).

### 4.7. Statistics

The data are reported as the mean ± standard error of the mean (s.e.m.). Unless otherwise indicated, the numbers (n) refer to oocytes used for each experiment and represent the fundamental unit for data collection and statistical analysis. Before data analysis, normal distribution was assessed with the Shapiro–Wilk test, and according to the result, parametric (Student’s *t*-test), non-parametric (Wilcoxon signed rank test, Mann–Whitney rank sum test), and chi-squared tests were performed using Sigmaplot 12 software. Differences between the two data sets were considered significant when *p* < 0.05, two-tailed.

## 5. Conclusions

Our findings suggest that membrane microtransplantation into *Xenopus* oocytes can be used as a robust, powerful tool to recover and investigate patient-derived cancer cell membranes from frozen biopsies. This approach could provide a methodological framework and translational approach for conducting molecular, functional, and imaging studies, ultimately enabling the reutilization of otherwise inaccessible clinical samples. Importantly, the observed impaired chloride channel activity may represent a pathophysiological hallmark of colon cancer and a potential therapeutic target.

## Figures and Tables

**Table 1:** Mortality assay in *Xenopus* oocytes injected with cell membranes from colon cancer.

	Survival Rate (24 h) ^1,3^	Survival Rate (48 h) ^2,3^
Sham injection	35/40 (87.5%);	33/41 (80.4%);
**50%**	7/19 (36.8%);	5/19 (26.3%);
**16.7%**	12/25 (48.8%);	12/25 (48.8%);
**4.2%**	7/8 (87.5%);	7/8 (87.5%);

^1^ Oocytes were obtained from two frogs. ^2^ Oocytes were obtained from four frogs. ^3^ Data were obtained from Patient 1.

**Table 2:** Antibody characteristics.

Antibody	Batch	Reference	Manufacture	Dilution
CEA	131	ND	NIH	1/500
E-Cadherin	NCH3-38	IR059	Agilent	RTU
Cytokeratin	CAM 5.2	349205	BD	RTU

**Table 3:** Calcium-activated chloride currents amplitude in cancer and corresponding normal tissues (TT; NT).

	NT		TT	
	Mean; Range(nA)	Cell Number (n)	Mean; Range (nA)	Cell Number (n)
Patient 1	102.9 ± 1.4 (99.0–108.0)	6	6.0 ± 1.2 (1.5–9.3)	7
Patient 2	85.4 ± 2.6 (79.0–88.0)	4	2.7 ± 0.6 (1.5–3.5)	3
Patient 3	86.6 ± 2.3 (83.7–91.0)	3	2.4 ± 0.4 (1.6–3.1)	4

NT = normal tissue (histologically confirmed normal mucosa). TT = tumor tissue.

## Data Availability

The data used to support the findings of this study are provided within this article. Further information can be provided by the corresponding author upon request.

## Supplementary Materials

The following supporting information can be downloaded at: https://www.mdpi.com/article/10.3390/ijms27031298/s1.
