# Supplementary material for: Reanimation of Stored Tissue Biopsies: A Functional Study and Translational Approach
Source: Int J Mol Sci. 2026 Jan 28;27(3):1298. doi: 10.3390/ijms27031298 (PMC12898366; doi:10.3390/ijms27031298)
Supplement: Supplementary file 1 [file ijms-27-01298-s001.zip › ijms-4089498-supplementary.pdf]

		Injected cells from colon tumor		Cells injected with TLE or ALS tissues	
**Neurotransmitters**	**Concentration (mM)**	**Mean (nA)**	**N**	**Mean ± SEM (Range;nA, Sample)**	**N**
GABA	0.5	-	8	-26.0± 4.3 (10.4-38, TLE)	8
ACh	0.5	-	5	-14.6± 3.0 (4-22.4, ALS)	5
Gly	0.5	-	9	-6.3± 0.9 (3.1-10.5, TLE)	9
Glutamate	1	-	7	-47.3± 2.6 (35.7-55.2, TLE)	7

Supplementary Table 1. The table shows the results from a set of experiments to evaluate the mean responses (Mean ± SEM, nA) of cells injected with colon tumor membranes diluted at 50% (dil 1:2 with membrane buffer) and positive controls (TLE or ALS. For each group, N indicates the number of cells tested and the mean current response (nA). Values in parentheses represent the response range (nA) and the tissue type, either TLE or ALS. These tissues were selected as positive controls since previous experiments already confirmed the presence of neurotransmitter-evoked responses in these cases (Palma et al., 2005; 2016).

Data were collected at 48 h post-injection using oocytes from 3 donor frogs (3 independent experiments). Each oocyte represents a single measurement. A dash ("–") indicates data that was not measured, as cells injected from colon cancer did not produce functional responses; otherwise, positive controls have been extensively studied, recorded responses on functional receptors. ALS, amyotrophic lateral sclerosis; TLE, temporal lobe epilepsy.
